# The *Neisseria gonorrhoeae* Vaccine Candidate NHBA Elicits Antibodies That Are Bactericidal, Opsonophagocytic and That Reduce Gonococcal Adherence to Epithelial Cells

**DOI:** 10.3390/vaccines8020219

**Published:** 2020-05-13

**Authors:** Evgeny A. Semchenko, Christopher J. Day, Kate L. Seib

**Affiliations:** Institute for Glycomics, Griffith University, Gold Coast 4215, Australia; e.semchenko@griffith.edu.au (E.A.S.); c.day@griffith.edu.au (C.J.D.)

**Keywords:** *Neisseria gonorrhoeae*, gonococcus, *Neisseria* heparin binding antigen (NHBA), bactericidal activity, opsonophagocytic activity, infection blocking, functional activity, vaccine

## Abstract

Due to the continuing emergence of multidrug resistant strains of *Neisseria gonorrhoeae* there is an urgent need for the development of a gonococcal vaccine. We evaluated the gonococcal *Neisseria* heparin binding antigen (NHBA) as a potential vaccine candidate, in terms of its sequence conservation and expression in a range of *N. gonorrhoeae* strains, as well as its immunogenicity and the functional activity of antibodies raised to either the full length NHBA or a C-terminal fragment of NHBA (NHBA-c). The gene encoding NHBA is highly conserved and expressed in all *N. gonorrhoeae* strains investigated. Recombinant NHBA is immunogenic, and mice immunized with either NHBA or NHBA-c adjuvanted with either Freund’s or aluminium hydroxide (alum) generated a humoral immune response, with predominantly IgG1 antibodies. Antibodies generated by both NHBA and NHBA-c antigens promoted complement activation and mediated bacterial killing via both serum bactericidal activity and opsonophagocytic activity, with slightly higher titers seen for the NHBA-c antigen. Anti-NHBA was also able to block the functional activity of NHBA by reducing binding to heparin and adherence to cervical and urethral epithelial cells. These data suggest that the gonococcal NHBA is a promising vaccine antigen to include in a vaccine to control *N. gonorrhoeae*.

## 1. Introduction

The ongoing emergence of multidrug resistant strains of *Neisseria gonorrhoeae* is a major challenge to the management of the sexually transmitted infection gonorrhoea [1,2]. The World Health Organization [3], Centers for Disease Control [4], and Australian National Antimicrobial Resistance (AMR) Strategy [5] have prioritised *N. gonorrhoeae* as an urgent public health threat for which immediate action is needed. There are estimated to be more than 106 million cases of gonorrhoea worldwide each year [6] and infection rates are rising (e.g., over the past five years, there has been a 67% increase in cases in the USA [7] and an 80% increase in Australia [8]). The outcome of *N. gonorrhoeae* infection varies by site of infection and by sex [9], and includes asymptomatic and localised symptomatic infection, but if left undiagnosed or untreated can result in severe sequelae, such as pelvic inflammatory disease, pregnancy and neonatal complications, and infertility. Infection with *N. gonorrhoeae* also increases the risk of acquiring and transmitting HIV. Due to its high prevalence, the severe sequelae it can cause, and the increasing difficulty of treating multi-drug resistant strains of *N. gonorrhoeae*, there is an urgent need for the development of a vaccine to prevent infection.

There are various challenges to developing a gonococcal vaccine, including the high level of phase and antigenic variation of *N. gonorrhoeae* surface structures, and the fact that there is no protective immunity following infection, which means there are no established correlates of protection to guide preclinical vaccine studies [9,10]. However, there have been several recent advances that support the feasibility of gonococcal vaccine development. A recent observational study suggested that a vaccine against the closely related bacteria *Neisseria meningitidis*, the outer membrane vesicle (OMV) meningococcal B vaccine MeNZB, had an effectiveness of 31% against infection with *N. gonorrhoeae* [11]. A newer four-component meningococcal B vaccine, 4CMenB (marketed as Bexsero) that contains the MeNZB OMV component plus three recombinant protein antigens, has been shown to induce cross reactive antibodies to *N. gonorrhoeae* proteins including the *Neisseria* heparin binding antigen (NHBA) [12].

The *N. meningitidis* NHBA (previously called GNA2132) is a component of 4CMenB, present as a NHBA-GNA1030 fusion protein [13]. The meningococcal NHBA (NHBA_Nm_) is a surface-exposed lipoprotein, that consists of three regions: an N-terminal region (up to residues 200–250) that is predicted to be intrinsically disordered and unfolded [14]; a central arginine-rich region that binds glycans including heparin, heparin sulfate and chondroitin sulfate [15,16,17], and a C-terminal region that folds as an anti-parallel β-barrel [14,18,19]. NHBA_Nm_ is relatively well conserved, although the N-terminal region contains several insertions/deletions between different meningococcal strains [14]. NHBA_Nm_ induces serum bactericidal antibodies against diverse *N. meningitidis* strains [16,20,21], and these antibodies are also opsonophagocytic [22,23] and are able to block adherence of *N. meningitidis* to epithelial cells [17]. The meningococcal NHBA is cleaved by NalP in some strains releasing the C-terminal region of the protein [14], however, *N. gonorrhoeae* does not express NalP (Neisseria autotransporter serine protease) [24]. The gonococcal homologue of NHBA (NHBA_Ng_) is conserved between *N. gonorrhoeae* strains, and shares 67% identity with the NHBA-2 peptide variant that is in 4CMenB [12,25]. We recently showed that NHBA_Ng_ is surface exposed and is recognized by antibodies from people vaccinated with 4CMenB [12]. We have also shown that NHBA_Ng_ binds to several glycans including heparin (K_D_, 4.4 nM) and chondroitin sulfate (K_D_, 73 nM), and is involved in interactions with both gonococcal and host cells [26]. Furthermore, a gonococcal *nhba* mutant has decreased cell aggregation and microcolony formation, and reduced survival in human serum and reduced adherence to human cervical and urethral epithelial cells, relative to the wild type strain [26]. However, the exact nature of gonococcal NHBA-mediated interactions with the host has not been determined and the host receptor(s) involved in adherence are unknown. 

In this study, we perform a detailed analysis of the sequence variation and expression of NHBA in *N. gonorrhoeae*, and investigate the level, type and functional activity of antibodies raised to NHBA_Ng_ to evaluate the potential of the full length protein and a C-terminal fragment of the protein as a gonococcal vaccine candidate. 

## 2. Materials and Methods 

### 2.1. Bacterial Strains and Growth Conditions 

*N. gonorrhoeae* strains 1291, FA1090, WHO G, WHO P and WHO X were used in this study. *N. gonorrhoeae* was grown on GC agar (Oxoid, Thermofisher Scientific, Waltham, MA, USA) with 1% (v/v) IsoVitaleX (Becton Dickinson, Franklin Lakes, NJ, USA) at 37 °C or 32 °C with 5% CO_2._ The majority of the gonococcal population used in assays were piliated and expressed opacity proteins as determined by visual inspection of colonies using phase contrast microscopy.

### 2.2. Sequence Analysis 

Sequences were aligned with MacVector (Apex, NC, USA), and the percentage of amino acid identity and similarity were calculated (BLOSUM90, threshold 0). The Neighbour-joining phylogenetic tree (best tree, uncorrect(“*p*”)) of NHBA variants was constructed with MacVector. The presence and conservation of *nhba* and the encoded NHBA protein between gonococcal strains was determined as at 19 September 2019 using the Basic Local Alignment Search Tool program (BLAST; National Library of Medicine, Bethesda, USA) with *nhba* from *N. gonorrhoeae* 1291 (GenBank Accession EEH61857.1; genome locus tag NGAG_00725) against 594 gonococcal genomes in GenBank and 5652 *N. gonorrhoeae* isolates in Neisseria Multi Locus Sequence Typing website (PubMLST; https://pubmlst.org/neisseria/). Previously established PubMLST nomenclature for NHBA (encoded by NEIS2109) was used, where every unique peptide sequence is assigned a unique identification number (e.g., NHBA_peptide 2 (NHBA-2) is in 4CMenB and NHBA_peptide 542 (NHBA-542) is in *N. gonorrhoeae* strain 1291).

### 2.3. Construction of N. Gonorrhoeae NHBA Mutant Strains

The *N. gonorrhoeae* 1291 *nhba* gene was amplified using 5′-ATGTTTAAACGCAGTGTGATTGC-3′ and 5′-TCAATCCCGATCTTTTTTGCCGGC-3′ primers and cloned into the pGEM-T easy vector (Promega). A kanamycin resistance gene (pUC4Kan; Amersham Biosciences, Piscataway, NJ, USA) was inserted into BamHI restriction site that was introduced into the middle of the *nhba* open reading frame using inverse PCR with 5′-ggatccCCGGCCGAGATTCCGCTGATTCC-3′ and 5′-ggatccGCGACCTCCTCGACCGTGCAGAAC-3′ primers (BamHI restriction enzyme sites introduced for subcloning of the kanamycin resistance gene into the *nhba* gene are shown in lower case). The *nhba::kan* construct was linearized with NcoI and transformed into *N. gonorrhoeae* 1291 to generate 1291 *nhba::kan* strain (ΔNHBA). The complemented strain (ΔNHBA_C) was generated by introducing the intact *nhba* gene (amplified using 5′-GGCATATGGCGGAAACAATA-3′ and 5′-TCAATCCCGATCTTTTTTGCCGGC-3′ primers) into the ΔNHBA strain using the complementation plasmid pCTS32 [27]. Successful deletion and subsequent complementation of the *nhba* gene was confirmed by PCR and Western blot.

### 2.4. Recombinant Protein Expression

Cloning and expression of the full length recombinant NHBA devoid of the predicted signal peptide was described previously [12]. For expression of the C-fragment of NHBA (NHBA-c), *E. coli* BL21 (DE3) was transformed with the pET19b plasmid containing cNHBA amplified from *N. gonorrhoeae* 1291 using primers 5′-ATTActcgagTCGCTTCCGGCCGAGATTCC-3′ and 5′-TGAAggatccCGGCATCAACATCAATC-3′ (XhoI and BamHI sites are shown in lower case, in the respective primers). Expression was induced by addition of 1 mM IPTG to OD_600_ 0.4 culture and incubation at 20 °C for 24 h. Protein was purified using TALON affinity resin (Clontech, Mountain View, CA, USA) as described previously [12].

### 2.5. Generation of Polyclonal Antibodies

Groups of five 4–5 week old female BALB/c mice (Animal Resources Centre, WA, Australia) were immunized subcutaneously with 25 µg of recombinant protein with Freund’s adjuvant (Merck, Darmstadt, Germany; Freund’s complete adjuvant (FCA) on day 0 and Freund’s incomplete adjuvant (FIA) subsequently) or with aluminium hydroxide (Alhydrogel; InvivoGen, San Diego, CA, USA) on days 0, 21, 28 and 42. Terminal bleeds were collected on day 56 and serum collected via centrifugation. Pre-immune serum was collected from each mouse prior to immunization. This study was carried out in accordance with the recommendations of the Australian Code for the Care and Use of Animals for Scientific Purposes, and with approval from the Griffith University Animal Ethics Committee (GLY/21/17/AEC). 

Polyclonal NHBA antibodies were purified from mouse sera using affinity chromatography with recombinant NHBA. NHBA was coupled to N-Hydroxysuccinimidyl-Sepharose 4 Fast Flow (Merck, Darmstadt, Germany) using manufacturer’s instructions and incubated with mouse sera diluted 1/2 with PBS. Bound antibodies were eluted with 0.1 M glycine buffer (pH 3.0). Eluted samples were buffer exchanged into PBS using Amicon-Ultra centrifugal spin unit (Merck, Darmstadt, Germany). Antibody concentration was determined with BCA (Thermofisher Scientific, Waltham, MA, USA).

### 2.6. Enzyme-Linked Immunosorbent Assays (ELISAs)

ELISAs were performed in triplicate using 96-well MaxiSorp (NUNC) plates, coated with 100 ng of purified recombinant protein in 100 μL of coating buffer (0.5 M carbonate/bicarbonate buffer, pH 9.6) for 1 h at room temperature as described previously [12,28,29]. Pooled sera from five mice per group were used for the primary antibody, and secondary antibody as specified in the results (polyclonal rabbit anti-mouse IgG HRP (Merck, Darmstadt, Germany) or goat anti-mouse IgG1, IgG2a, IgG2b, IgG3, or IgM HRP (Thermofisher Scientific, Waltham, MA, USA). The ELISA titer is the highest serum dilution with absorbance at 450 nm greater than mean negative (all reagents excluding primary antibody) + 3 standard deviations.

### 2.7. Serum Bactericidal Activity (SBA) and Opsonophagocytic Killing (OPA) Assays

SBA and OPA assays were performed as described previously [28,30]. Briefly, approximately 1 × 10^3^ colony forming units (CFU) of *N. gonorrhoeae* were incubated in serial dilutions of heat-inactivated (56 °C, 60 min) anti-NHBA or pre-immune mouse sera (pooled sera from five mice per group) for 15 min at 37 °C. The SBA assay was initiated by adding the complement source (10% (v/v) normal human serum pre-absorbed with *N. gonorrhoeae* [28]), followed by incubation at 37 °C, 5% CO_2_ for 30 min. Complement at the concentration used is not bactericidal on its own to wild type *N. gonorrhoeae*. The NHBA deletion mutant strain (ΔNHBA) is sensitive to human serum [27], including 10% serum pre-absorbed with *N. gonorrhoeae* (Figure S1), and as such was not tested in SBA assays. The OPA assay was initiated by adding the complement source and ~1 × 10^5^ polymorphonuclear leukocytes (PMNs), followed by incubation at 37 °C, 5% CO_2_ for 90 min. Serial dilutions of the contents of each well were plated on GC agar and grown overnight. The SBA or OPA titer is the highest antibody dilution which induced more than 50% killing in the assay. The presence of complete complement in the OPA assay means that some killing may also be due to bacterial lysis via SBA. Statistical analysis was performed using one-way analysis of variance (ANOVA) and two-tailed Student’s *t*-test. Each experiment was performed three times, with triplicate samples in each experiment. Human sera and PMNs were collected from healthy male and female adult volunteers with approval by the Griffith University Human Ethics Committee (HREC 2012/798) and with written informed consent by the donors.

### 2.8. Flow Cytometry Analysis

Antibody binding to *N. gonorrhoeae* and C3 fragment deposition was measured using flow cytometry as described previously [30]. Briefly, *N. gonorrhoeae* 1291 (~1 × 10^7^ CFU) was pre-incubated with 1:100 dilution of heat-inactivated mouse sera or 70 µg/mL of purified NHBA antibodies in HBSS^+^ (Hank’s Balanced Salt Solution containing 0.15 mM CaCl_2_ and 0.5 mM MgCl_2_ and 1% BSA (w/v)). Antibody treated bacteria were washed and incubated with 1:200 dilution of Alexa Fluor 488 conjugated anti-mouse IgG (Thermofisher Scientific, Waltham, MA, USA) or with 5% normal human serum pre-absorbed with *N. gonorrhoeae* for 15 min at 37 °C, after which C3 fragments were detected by incubating bacteria with 1:200 dilution of FITC conjugated anti-human C3c antibody (BioRad, California, USA). Data were acquired using CyAn ADP flow cytometer (Beckman Coulter, Brea, CA, USA) and analysed using FlowJo (BD BioScience, San Jose, CA, USA).

### 2.9. Surface Plasmon Resonance (SPR)

SPR competition assays were performed using a Pall Pioneer FE. Competition assays were performed as previously described [28] using NextStep injections in the OneStep assay builder. Pre- and post-immune NHBA mouse sera were used as the first injection (A) and heparin as the second injection (B). Binding of heparin (maximum OneStep concentration of 50µM) to NHBA was compared with and without serum, and with 1:200 dilution of pre- or post-immune serum. Data was collected using the Pioneer Software package (ForteBio, Fremont, CA, USA) and analysed using Qdat analysis software (ForteBio, Fremont, CA, USA). The percentage blocking was calculated based on the relative RMax of the heparin injection without serum (injection A = buffer; B = heparin) versus the serum subtracted (injection A = pre/post-immune serum; B = buffer) binding of heparin in the presence of serum (injection A = pre/post-immune serum; B = heparin).

### 2.10. Epithelial Cell Adherence Assays

Gonococcal adherence assays were performed as described previously [29] with the following modifications. Briefly, monolayers of tCX [31] and tUEC [32] cells (provided by M.A. Apicella, University of Iowa, Iowa City, USA) were infected (10 min at 37 °C) with approximately 1 × 10^5^ CFU that was initially pre-incubated with serial dilutions of heat-inactivated mouse sera (pooled sera from five mice per group) for 30 min at room temperature. Following the infection, cell monolayers were washed three times with warm HBSS to remove non-adherent bacteria, cells lysed in 1% saponin and well contents then plated onto GC agar. Results were calculated as the mean CFU from three replicate wells and presented as percentage of adherent bacteria relative to no antibody control. Statistical analysis performed with ANOVA and two-tailed Student’s *t*-test. Each experiment was performed three times.

## 3. Results

### 3.1. NHBA Is Highly Conserved in N. Gonorrhoeae 

**Figure 1:**
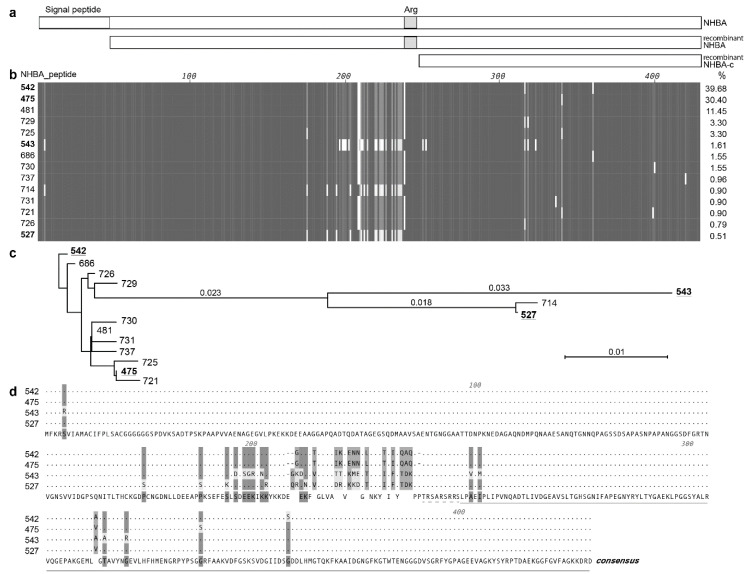
Overview of the gonococcal NHBA. (**a**) Schematic of the NHBA protein from *N. gonorrhoeae strain* 1291, showing the signal peptide region (open box) and the arginine rich region (Arg; grey box). The recombinant proteins used in the study are also shown, the mature NHBA (NHBA; lacking the predicted signal peptide) and the C-terminal fragment of NHBA (NHBA-c). (**b**) An alignment of the amino acid sequences of the 14 main NHBA variants of *N. gonorrhoeae*, with amino acids that are identical between all variants shown as a dark gray vertical line, amino acids conserved between most variant shown as light gray, mismatches or gaps shown as white. The NHBA peptide number is shown on the left and the % of isolates in PubMLST that contain this variant on the right. (**c)** Neighbour-joining phylogenetic tree of the 14 main NHBA variants. The four NHBA variants present in strains used in this study are underlined. (**d**) Amino acid alignment of the four NHBA variants present in strains used in this study. Matches to the consensus sequence (shown on the bottom line) are indicated by dots. The arginine rich region is indicated by a dashed line, and the NHBA-c fragment is indicated by a line.

We have previously shown that NHBA is conserved in *N. gonorrhoeae* [12] and here we further examine the sequence variants of NHBA in available gonococcal isolates and genome sequences. A blastn search with the 1281 nucleotide *nhba* gene from *N. gonorrhoeae* strain 1291, which encodes the 427 amino acid NHBA (Figure 1a), against the available gonococcal genomes in GenBank revealed that *nhba* is present in all 594 genomes, with 94.1–100% nucleic acid identity. A similar blastn search against the PubMLST database revealed the presence of the *nhba* gene in 4424 isolates with 85.1–100% identity. The 1228 isolates that did not have a match to *nhba* in this BLAST search were also missing annotated 16S and *porB* genes, indicating that incomplete sequences are available for these isolates. This confirms that *nhba* is widely distributed and highly conserved in a temporally and geographically diverse panel of gonococcal strains that were collected between 1960 and 2020 from >60 different countries.

As at 6 April 2020, there are 42 unique NHBA_peptide variants in the 3546 *N. gonorrhoeae* isolates that have an annotated NHBA protein in the PubMLST database. These variants share 97.5–100% amino acid identity. There are two predominant NHBA variants that are present in 70.3% of PubMLST isolates, NHBA-542 (present in 39.7% of strains, including *N. gonorrhoeae* 1291) and NHBA-475 (present in 30.4% of strains, including *N. gonorrhoeae* WHO P and WHO X). Overall, one of 14 main NHBA variants is present in 97.8% of isolates, while the remaining 28 NHBA peptide variants are rare, being present in between 1–10 isolates (Table S1). Alignment of these 14 most common variants indicates that the N- and C-terminals have the highest level of conservation, with a variable central region present upstream of the arginine rich region (Figure 1b, variants arranged in order of decreasing abundance). The phylogenetic relatedness of these NHBA variants is shown in Figure 1c, and a panel of strains representative of NHBA diversity were used in subsequent assays. Given the sequence conservation of the C-terminal (Figure 1b), that the structure of the meningococcal NHBA C-terminal region has been characterized [15,18,19] and that the C-terminal region is more likely to be exposed and accessible to vaccine induced antibodies, we focused our subsequent investigation on both the recombinant full length NHBA and a C-terminal NHBA fragment (NHBA-c) (Figure 1a).

### 3.2. The Recombinant Full Length NHBA and the C-Terminal NHBA Fragment Are Immunogenic and Induce Antibodies that Recognize NHBA Variants from a Range of Gonococcal Strains

**Figure 2:**
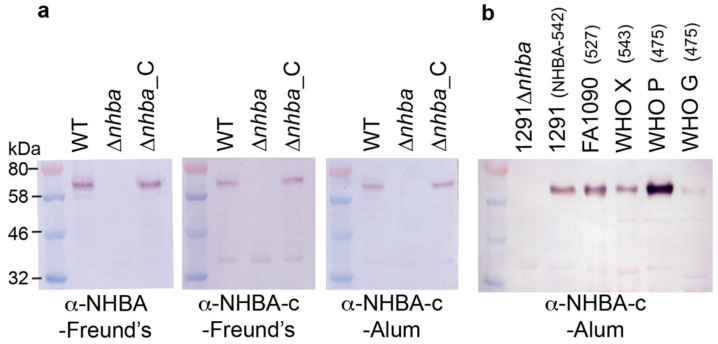
Expression of NHBA in a panel of gonococcal strains. Western blot analysis of NHBA expression in (**a**) *N. gonorrhoeae* 1291 wild type (WT), *nhba::kan* mutant (ΔNHBA) and complemented (ΔNHBA_C) strains; and (**b**) the gonococcal strains used in SBA and OPA assays. The NHBA peptide variant of strain is indicated in brackets. The sera used are indicated below the blots. All blots derived from the same experiment and were processed in parallel. Full SDS-PAGE gels and Western blots are shown in Figure S2.

To examine the immunogenicity of the gonococcal NHBA, sera from mice immunized with the recombinant full length NHBA plus Freund’s adjuvant or the NHBA-c fragment plus Freund’s or aluminium hydroxide (alum) were assessed by ELISA and Western blot. Using whole-cell ELISA, we show that both NHBA and NHBA-c mouse sera can detect native NHBA on the surface of *N. gonorrhoeae* wild type (WT) and the NHBA complemented (ΔNHBA_C) strains, with significantly reduced titers for the NHBA mutant strain (ΔNHBA) (Table 1). Analysis of NHBA antisera by Western blotting against whole cell lysates of *N. gonorrhoeae* wild type and the mutant confirmed that the antisera specifically recognizes NHBA (Figure 2a, Figure S2a,b). The expression of NHBA and the cross-reactivity of the NHBA antisera in a panel of *N. gonorrhoeae* strains was confirmed by Western blot analysis (Figure 2b, Figure S2c,d). NHBA expression varied between strains, and high, medium and low NHBA expressers were used in subsequent assays.

ELISA with the recombinant NHBA indicated the presence of a dominant IgG1 isotype response in mice immunized with NHBA (Table 1, Figure S3). However, the ratio of isotypes and subclasses differed between the different formulations, with pooled NHBA-Freund’s having higher levels of IgG3 and lower levels of IgG2a and IgG2b (IgG1 > IgM > IgG3 > IgG2b > IgG2a) than NHBA-c-Freund’s (IgG1 > IgM = IgG2b > IgG2a > IgG3) and NHBA-c-alum (IgG1 > IgM > IgG2b > IgG2a > IgG3). Overall, the ELISA and Western results confirm that the gonococcal NHBA is immunogenic and that anti-NHBA antisera can recognize NHBA on the surface of several *N. gonorrhoeae* strains that express different NHBA variants.

### 3.3. NHBA Antibodies Promote C3-Fragment Deposition

**Figure 3:**
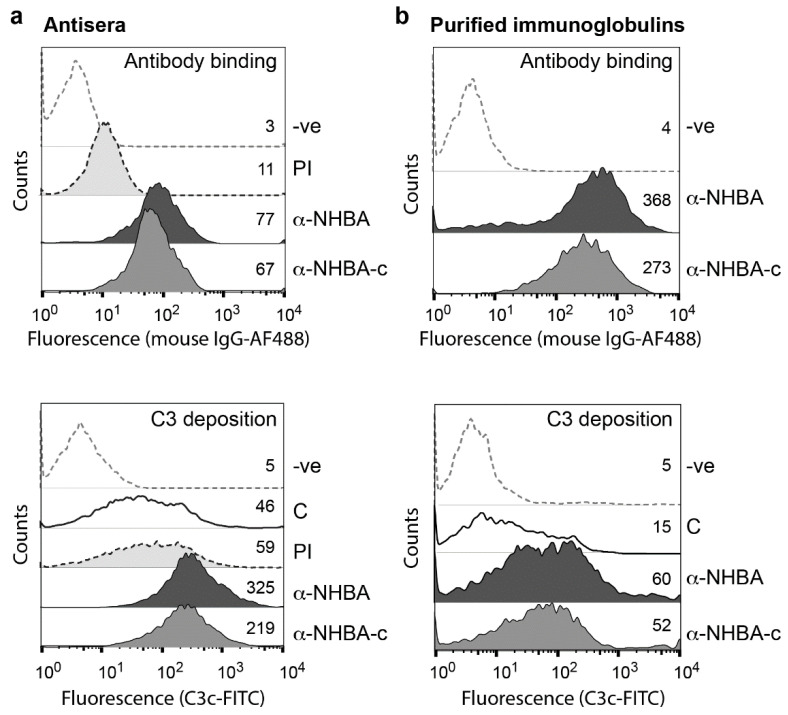
Antibody binding and complement activation as measured by fragment deposition on *N. gonorrhoeae.* Flow cytometry of antibody binding and antibody-mediated C3-fragment deposition on the surface of *N. gonorrhoeae* 1291, in the presence of (**a**) polyclonal antisera or (**b**) purified immunoglobulins from mice immunised with either NHBA-Freund’s or NHBA-c-Freund’s. Values represent geometric mean fluorescence of antibody binding to *N. gonorrhoeae* cells and C3-fragment deposition. Secondary antibody only (-ve), pre-immune (PI) mouse sera and complement only (C) controls are included. Sera and purified immunoglobulins used are from a pool of mouse sera from five mice per group. Experiments were done at least three times and a representative result is shown. In all panels, the geometric mean fluorescence of α-NHBA and α-NHBA-c are significantly above the PI and C controls (Student’s *t*-test *p* < 0.001).

To investigate if NHBA antisera promote activation of the complement cascade, C3-fragment deposition onto the surface of *N. gonorrhoeae* was investigated using flow cytometry. NHBA-Freund’s and NHBA-c-Freund’s mouse sera, as well as purified NHBA-specific immunoglobulins from these sera were tested, all of which bind *N. gonorrhoeae* strain 1291 as evidenced by an increase in mean fluorescence intensity relative to the pre-immune sera or the control treated bacteria (Figure 3a,b top panel). Bacteria incubated with human complement plus either the whole sera or purified immunoglobulins had markedly increased C3-fragment deposition, relative to the complement only control (7.1 and 5.2-fold increase, respectively, for NHBA; 4.8 and 4.7-fold increase, respectively, for NHBA-c; Figure 3a,b bottom panel). 

### 3.4. NHBA Antibodies Have Bactericidal and Opsonophagocytic Activity

**Figure 4:**
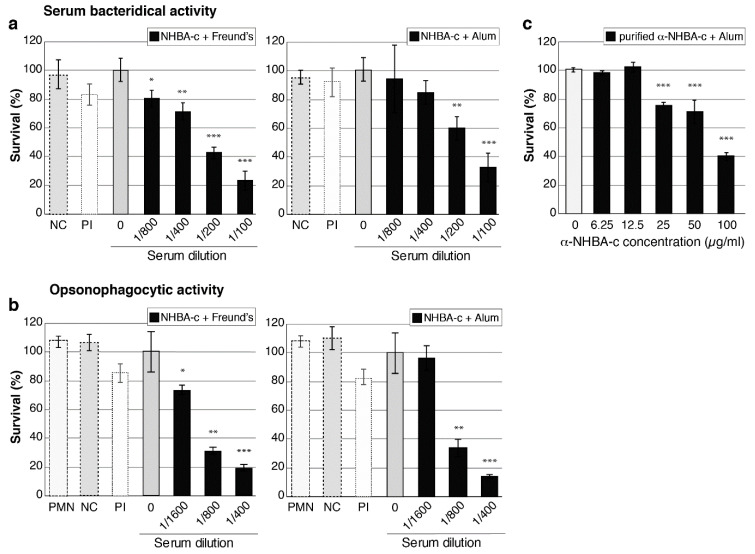
Serum bactericidal activity and opsonophagocytic activity of anti-NHBA antibodies. (**a**,**c**) Serum bactericidal activity (SBA) of anti-NHBA serum. The survival of *N. gonorrhoeae* strain 1291 in the presence of normal human serum as a source of complement and 2-fold dilutions of heat-inactivated mouse sera is shown. Sera are either: (**a**) anti-NHBA-c serum plus adjuvant (Freund’s or alum) compared to no serum (0) and pre-immune (PI) control sera. A ”no complement” control (NC) is also shown (bacteria incubated with 1/100 dilution of mouse sera only); or (**c**) purified anti-NHBA antibodies from NHBA-c-alum serum. (**b**) Opsonophagocytic activity (OPA) of anti-NHBA serum. The survival of *N. gonorrhoeae* strain 1291 in the presence of human polymorphonuclear leukocytes (PMNs), normal human serum and mouse sera are shown, as in (**a**) above. The ”no complement” control (NC) is shown (bacteria incubated with 1/400 dilution of mouse sera only), as well as a ”PMN only” control (PMN) (bacteria incubated with PMNs but no mouse sera and no complement). For a–c, data represent the average survival for triplicate samples relative to the result obtained with the untreated wild-type strain (0) (the untreated wild type, set at 100%, represent 2.5 × 10^3^, 1.6 × 10^3^ and 3.5 × 10^3^ colony forming units for a–c, respectively). Error bars represent ± 1 standard deviation. A two-tailed Student’s *t*-test was used to compare survival relative to the no serum (0) untreated wild type; *, *p* < 0.05 **, *p* ≤ 0.01, ***, *p* ≤ 0.001. Statistical analysis was also performed for (c) using one-way analysis of variance (ANOVA; *p* < 0.0001) and Dunnett’s multiple comparison test (*p* > 0.9 for untreated wild-type control group (0) vs. 6,25 or 12.5; *p* ≤ 0.0001 for 0 vs. 25, 50 or 100 µg/mL).

The ability of NHBA and NHBA-c antibodies to mediate complement-dependent lysis and opsonophagocytic killing of *N. gonorrhoeae* was tested using serum bactericidal activity (SBA) and opsonophagocytic killing (OPA) assays, respectively. Five gonococcal strains containing different NHBA variants and with variable NHBA expression levels were tested. For SBA assays, *N. gonorrhoeae* was incubated with NHBA or NHBA-c mouse sera before active source of human complement was added and bacterial survival measured. Both NHBA-Freund’s and NHBA-c-Freund’s sera elicited serum bactericidal activity in concentration dependent manner with SBA titers ranging from 100 to 1600 (compared to pre-immune sera titers < 50) (Table 2, Figure 4a). For OPA assays, *N. gonorrhoeae* opsonised with NHBA or NHBA-c antibodies and incubated in presence of human complement and human PMNs were killed in dose-dependent manner, with OPA titers ranging from 100 to 6400 (compared to pre-immune sera titers < 50) (Table 2, Figure 4b). Sera raised to NHBA formulated with an adjuvant that is frequently used in human vaccines alum (NHBA-c-alum) also induced SBA and OPA killing of *N. gonorrhoeae,* with titres similar for those seen by NHBA-c-Freund’s sera (Table 2, Figure 4a,b). The purified NHBA immunoglobulins from mice immunised with NHBA-c-alum mediated concentration dependent SBA killing (Figure 4c). Furthermore, no killing is seen in the NHBA-c-alum sera that has been depleted of anti-NHBA antibodies (Figure S4), confirming the specificity of the immune response for NHBA.

### 3.5. NHBA Antibodies Reduce NHBA Binding to Heparin, and Gonococcal Adherence to Host Cells

**Figure 5:**
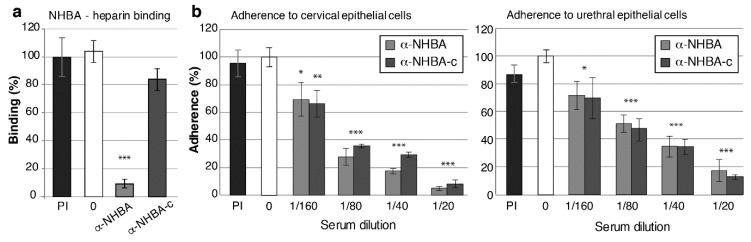
Functional blocking activity of NHBA antisera against *N. gonorrhoeae.* (**a**) Blocking of NHBA-heparin interactions using α-NHBA antibodies. Surface plasmon resonance (SPR) analysis of NHBA-heparin interactions was performed in the absence of sera (0; white) or in the presence of pre-immune sera (PI; black), α-NHBA sera (light grey) or α-NHBA-c sera (dark grey). Data represents the mean NHBA-heparin binding (+/− 1 standard deviation) for triplicate samples, as a percentage of binding in the absence of antibody (the no antibody control (white) set at 100%). (**b**,**c**) Blocking of *N. gonorrhoeae* adherence to epithelial cells. α-NHBA and α-NHBA-c serum-treated gonococci had significantly reduced adherence to (**b**) cervical and urethral epithelial cells at all tested concentrations (p < 0.05, calculated using a two-tailed Student’s *t*-test) relative to the untreated control (0; white). Pre-immune sera (PI, black), did not affect bacterial adherence (*p* > 0.05). Sera used are a pool of mouse sera from five mice per group. Results are shown as average percentage of adherent bacteria from triplicate serum-treated samples relative to no antibody control (result for no antibody controls set at 100% are 4.33 × 10^3^ and 1.37 × 10^3^ adherent colony forming units per monolayer for cervical and urethral cells, respectively). Error bars denote ± 1 standard deviation. Experiments were performed twice with triplicate samples and representative results are shown.

To investigate whether NHBA and NHBA-c antisera can inhibit the functional role of NHBA, we conducted surface plasmon resonance (SPR) based competitive-binding experiments with recombinant NHBA and its predicted substrate heparin, in the presence and absence of NHBA antisera. In the absence of antisera, gonococcal NHBA binds heparin. Pre-immune serum had no effect on the ability of heparin to interact with NHBA (*p* = 0.65) but serum from mice immunised with full length NHBA reduced heparin binding by 90% (*p* = 0.00002) (Figure 5a, Figure S5). However, the NHBA-c serum was unable to significantly inhibit the interaction between heparin and NHBA (16% reduction in binding; *p* = 0.11) (Figure 5a, Figure S5).

Gonococcal NHBA is involved in adherence to human cervical and urethral epithelial cells [26], therefore, we investigated whether NHBA and NHBA-c antisera can reduce adherence. In vitro infection assays were performed with transformed cervical (tCX) and urethral (tUEC) epithelial cells with *N. gonorrhoeae* that was pre-incubated with antisera. NHBA and NHBA-c sera, but not the pre-immune sera, are able to reduce adherence to both tCX and tUEC in a concentration dependent manner, relative to the no antibody control. For example, a 1:20 dilution of NHBA sera decreases gonococcal adherence 19- and 6-fold in tCX and tUEC cells, respectively. Similarly, a 1:20 dilution of NHBA-c antisera reduces bacterial adherence 8- and 5-fold in tCX and tUEC cells, respectively (Figure 5b). 

## 4. Discussion

In light of the threat of antimicrobial resistant *N. gonorrhoeae,* there is an increasing need for the identification and characterization of potential vaccine candidates to aid development of a gonococcal vaccine. Here we characterize the gonococcal NHBA and show that it is widely distributed and conserved in geographically and temporally diverse *N. gonorrhoeae* strains, and that antibodies raised to either the full length NHBA, or a C-terminal fragment of NHBA, mediate bactericidal and opsonophagocytic killing. These antibodies can also reduce adherence of *N. gonorrhoeae* to human epithelial cells and inhibit NHBA’s glycan-binding activity. There is currently no known correlate of protection for *N. gonorrhoeae* [9]*,* however the ability of NHBA to elicit antibodies that are able to kill *N. gonorrhoeae* via two conventional immune killing mechanisms, as well as mediate functional blocking of an important stage in infection, supports its potential to be used in a gonococcal vaccine.

The gonococcal NHBA is highly conserved, with ≥97.5% amino acid identity in the *N. gonorrhoeae* strains investigated to date, and with the majority of strains expressing one of a limited number of NHBA variants (e.g., 70.3% expressing one of two main variants, 91.3% expressing one of seven variants). NHBA expression is variable between strains, even between strains expressing the same NHBA variant (e.g., WHO X and WHO P (NHBA-475)), and NHBA expression will need to be investigated in a large collection of isolates from around the world. However, we show that antisera raised to NHBA variant 542 (from *N. gonorrhoeae* strains 1291) was cross reactive and able to kill strains expressing homologous and heterologous NHBA variants, and strains with high, medium and low NHBA expression in a dose-dependent manner in both SBA and OPA assays. The specific role of anti-NHBA in the anti-gonococcal immune response was confirmed as seen by dose-dependent SBA killing with purified NHBA-specific immunoglobulins and the absence of killing in sera depleted of anti-NHBA antibodies. Although *N. gonorrhoeae* and *N. meningitidis* strains contain different predominant NHBA variants [12], the cross protection seen for gonococcal strains is consistent with findings for NHBA of *N. meningitidis* where immune reactivity of antibodies to NHBA-2 (found in 4CMenB) was seen for 99.5% of strains circulating in the United States (442 strains), irrespective of their NHBA variant [33]. In addition to NHBA sequence and expression level, a combination of other factors is also likely to contribute to the SBA and OPA titres seen for different strains, including intrinsic strain features that can affect complement regulation and susceptibility of each strain to human serum used as a complement source. Similar intrinsic differences between strains have also been reported for anti-NHBA [34] and anti-fHbp bactericidal activity against *N. meningitidis* [35,36]. The SBA titres ranged from 50 to 1600 for the different vaccine antigens and formulations against different strains. A wide range of SBA titers have been reported for other antigens tested against various *N. gonorrhoeae* strains, for example MetQ and MsrAB have SBA titres of 320 and 100, respectively, against strain 1291 [28,30], NGO0690 has a SBA titre of 10 against strain FA1090, and NGO1701 has a SBA titre of 40 against strain F62 [37]. However, assays are not standardised which makes direct comparisons difficult. It is important to note that the SBA titres and the OPA titres for strains FA1090 (NHBA-527) and WHO G (NHBA-475) were similar, and since the terminal complement pathway in the OPA assay is intact, killing in the OPA assay of these two strains may be attributed to killing through terminal complement mediated lysis. 

Antibodies to the full length NHBA or a C-terminal fragment of NHBA can reduce gonococcal adherence to cervical and urethral epithelial cells. Adherence was reduced by up to 80% when ~10^5^ bacteria were added to cell monolayers in the presence of NHBA antisera. This is above the estimated infectious dose for gonococcal infection (e.g., ID50 of the male urethra ~ 2.3 × 10^3^ CFU [38]), which suggests that vaccine-induced antibodies could reduce the level of initial host colonization by *N. gonorrhoeae*. NHBA interacts with several glycans [26] and antibodies to the full length NHBA, but not NHBA-c, and were able to block interactions with heparin. In *N. meningitidis* the central arginine-rich region of NHBA is required for heparin binding [16], and antibodies to the arginine-rich region of the full length gonococcal NHBA may be responsible for reduced heparin binding. However, the arginine-rich region is absent from the C-terminal fragment of NHBA, which may explain why antibodies to NHBA-c do not alter heparin binding. The host receptor (s) required for gonococcal NHBA-mediated adherence are unknown, and while heparin may be involved, it is likely not the only factor, given that NHBA-c antisera reduces bacterial adherence to a similar level as NHBA antisera. 

The NHBA and the NHBA-c fragment were immunogenic in mice when adjuvanted with either Freund’s or aluminium hydroxide. Overall, an IgG1-dominant antibody response was elicited in all cases indicating a predominantly Th2 type antibody response, although varying patterns of other isotypes and subclasses were seen for the different antigen and adjuvant combinations. Furthermore, although NHBA-c produced lower total IgG titres compared to the full length NHBA, it elicited similar or higher SBA and OPA titres against most strains in the panel investigated. Immunoglobulin isotypes and subclasses are known to differ in their ability to activate complement and mediate bactericidal and opsonophagocytic activity, although this ability varies between antigenic targets. For example, mouse monoclonal antibodies targeting the *N. meningitidis* PorA antigen have a hierarchy of IgG3 >> IgG2b > IgG2a >> IgG1 for serum bactericidal activity (50% killing seen with 6 ng/µL 187,G-12 (IgG3) compared to 2500 ng/µL of 221,A-7 (IgG1)) and IgG3 > IgG2b = IgG2a >> IgG1 opsonophagocytic activity (50% killing seen with 80 ng/µL 187,G-12 (IgG3) compared to 3200 ng/µL of 221,A-7 (IgG1)) [39]. Similarly, mouse antibodies against the *N. gonorrhoeae* antigen MsrA/B have higher titers of IgG2a, IgG2b and IgG3 when adjuvanted by Freund’s compared to aluminium hydroxide, and the MsrA/B-Freund’s antisera, but not MsrA/B-alum antisera, mediated SBA and OPA killing of *N. gonorrhoeae* [30]. Our data suggest that IgG2a and IgG2b play a dominant role in the anti-NHBA SBA and OPA mediated killing of *N. gonorrhoeae*, as higher total levels of these antibodies were elicited by NHBA-c-Freund’s compared to NHBA-Freund’s. Furthermore, NHBA-c-alum elicited IgG2b > IgG2a > IgG3 and mediated SBA and OPA against all five gonococcal strains tested. This is distinct from MsrA/B-alum that did not elicit IgG2a, IgG2b or IgG3 and anti-MsrA/B-alum did not mediate killing of *N. gonorrhoeae* [30]. This difference in antibody levels and function may be antigen specific or may be associated with the different immunisation doses and schedules used in the different studies (NHBA, 25µg days 0, 21, 28 and 42 vs. MsrA/B 5µg on days 0, 21 and 28). Both Freund’s and alum were used as adjuvants in this study due to the variability in SBA and OPA killing of *N. gonorrhoeae* that has previously been seen for different antigen and adjuvant combinations, and with the aim to determine the key humoral immune components required for protection against *N. gonorrhoeae.* This is key for future identification of a correlate of protection for *N. gonorrhoeae* and development of a gonococcal vaccine. 

## 5. Conclusions

Overall, we describe several key features of NHBA that support its use as an antigen in a gonococcal vaccine, including its widespread distribution and conservation in gonococcal strains, and its ability to induce antibodies in mice that promote complement activation and mediate bacterial killing via both serum bactericidal activity and opsonophagocytic activity. We also highlight the potential to use the C-terminal fragment of NHBA as an optimized antigen that could be used alone, or in combination with other antigens.

## Figures and Tables

**Table 1:** Antigenicity of gonococcal NHBA and NHBA-c.

Sera ^1^	Total IgG ELISA Titre			ELISA Titre					
	Vs. Whole-Cells ^2^			Vs. Recombinant NHBA ^3^					
	WT	∆NHBA	∆NHBA_C	Total IgG	IgG1	IgG2a	IgG2b	IgG3	IgM
**NHBA-Freund’s**	128,000	16,000	128,000	40,960,000	81,920,000	25,600	51,200	102,400	409,600
**NHBA-c Freund’s**	64,000	8000	64,000	20,480,000	40,960,000	51,200	204,800	12,800	204,800
**NHBA-c alum**	32,000	4000	32,000	10,240,000	40,960,000	25,600	51,200	6400	102,400

^1^ Sera used are a pool of mouse sera from five mice per group. ^2^ Whole cell *N. gonorrhoeae* 1291 wild type (WT), *nhba::kan* mutant (∆NHBA), and complemented (∆NHBA_C) strains. The titres against *N. gonorrhoeae* strains were ≤4000 for pre-immune sera and for adjuvant only controls. ^3^ The total IgG titres against recombinant NHBA were ≤1600 for pre-immune sera and <10,000 for adjuvant only control. ELISAs were performed with triplicate samples on three occasions, and data shown is the mean from a single representative experiment.

**Table 2:** Serum bactericidal and opsonophagocytic titers of NHBA and NHBA-c mouse sera against five gonococcal strains.

Strain	NHBA Variant	NHBA Expression ^1^	Sera ^2^					
			NHBA Freund’s		NHBA-c Freund’s		NHBA-c alum	
			SBA	OPA	SBA	OPA	SBA	OPA
**1291**	**542**	++	100	400	200	800	100	800
FA1090	527	+++	100	100	100	100	50	100
WHO G	543	+	200	200	200	400	100	100
WHO X	475	++	1600	6400	1600	6400	1600	3200
WHO P	475	++++	200	800	200	800	200	400

^1^ NHBA expression level as determined by visual inspection of Western blots with whole-cell lysates (see Figure 2b, Figure S2c), and assigned as low (+), medium (++), high (+++) or very high (++++). ^2^ Sera used are a pool of mouse sera from five mice per group. SBA, serum bactericidal activity titre; OPA, opsonophagocytic titre (reciprocal of the lowest antibody dilution which induced more than 50% killing). The titres of pre-immune sera against *N. gonorrhoeae* strains were <50 in SBA and OPA assays.

## Supplementary Materials

The following are available online at https://www.mdpi.com/2076-393X/8/2/219/s1. Figure S1: Survival of *Neisseria gonorrhoeae* in human serum, Figure S2: Expression of NHBA in *N. gonorrhoeae*, Figure S3: Immunogenicity of NHBA, Figure S4: Serum bactericidal activity (SBA) of anti-NHBA serum, Figure S5: Surface plasmon resonance (SPR) analysis of NHBA-heparin interactions, Table S1: Distribution of NHBA peptide variants in *N. gonorrhoeae* isolates that have an annotated NHBA protein in the PubMLST database.
