# Supplementary material for: The Neisseria gonorrhoeae Vaccine Candidate NHBA Elicits Antibodies That Are Bactericidal, Opsonophagocytic and That Reduce Gonococcal Adherence to Epithelial Cells
Source: Vaccines (Basel). 2020 May 13;8(2):219. doi: 10.3390/vaccines8020219 (PMC7349534; doi:10.3390/vaccines8020219)
Supplement: Supplementary file 1 [file vaccines-08-00219-s001.pdf]

vaccines

Supplementary Material

The *Neisseria gonorrhoeae* vaccine candidate NHBA elicits antibodies that are bactericidal, opsonophagocytic and that reduce gonococcal adherence to epithelial cells

Evgeny A. Semchenko [1], Christopher J. Day [1] and Kate L. Seib [1,*]

[1] Institute for Glycomics, Griffith University, Gold Coast, QLD, Australia; e.semchenko@griffith.edu.au (E.A.S.); c.day@griffith.edu.au (C.J.D.); k.seib@griffith.edu.au (K.L.S.)

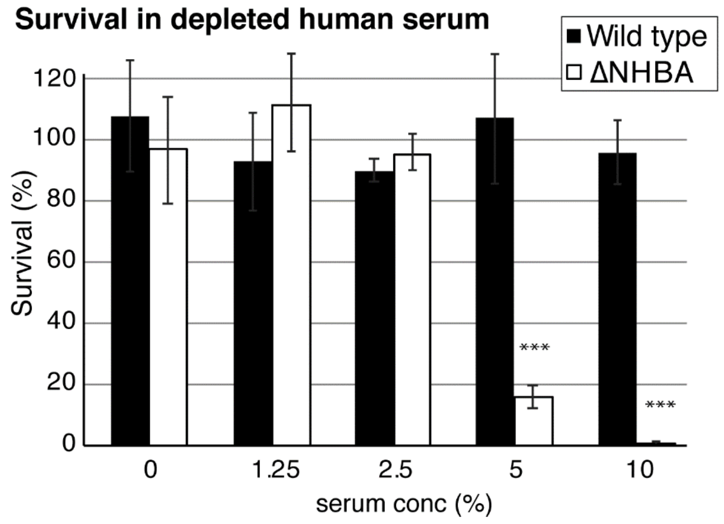

Figure S1. Survival of *Neisseria gonorrhoeae* in human serum. The survival of *Neisseria gonorrhoeae* 1291 wild type (WT) and NHBA knockout (ΔNHBA) strains after 30 minutes in 0-10% (vol/vol) human serum is shown. The human serum tested is normal human serum pre-absorbed with *N. gonorrhoeae* to remove any antibodies that cross react with *N. gonorrhoeae.* This depleted serum is used as a complement source in serum bactericidal activity (SBA) and opsonophagocytic killing (OPA) assays. Data represent the average survival for triplicate samples relative to the result obtained with the untreated strain (0) (the untreated WT, set at 100%, represents 2.8×10^3 colony forming units (CFU); untreated ΔNHBA, set at 100%, represents 2.5×10^3 CFU). Experiments were performed three times, and representative results are shown. A two-tailed Student's *t*-test was used to compare survival relative to the untreated control; ***, $p \leq 0.001$. There was no significant difference in survival of the WT in the % serum tested, relative to the untreated (0) control..

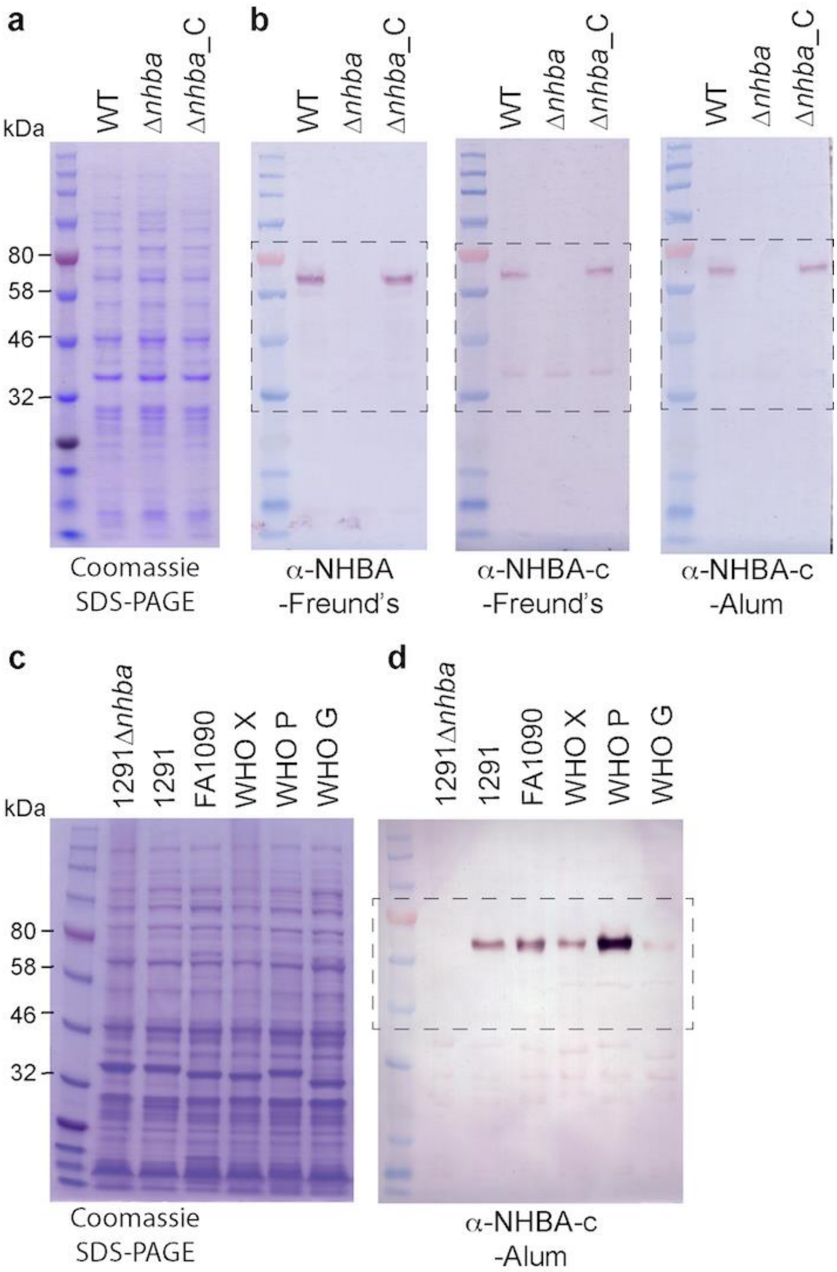

Figure S2. Expression of NHBA in *N. gonorrhoeae*. (**a**) Coomassie stained SDS-PAGE and (**b**) Western blot analysis of whole cell lysates of *N. gonorrhoeae* 1291 wild type (WT), *nhba::kan* mutant (ΔNHBA), and complemented (ΔNHBA_C) strains, probed with α-NHBA antibodies as indicated below the blots. The region of the Western blot shown in Figure 2a is boxed. (**c**) Coomassie stained SDS-PAGE and (**d**) Western blot analysis of whole cell lysates of a panel of *N. gonorrhoeae* strains. The region of the Western blot shown in Figure. 2b is boxed.

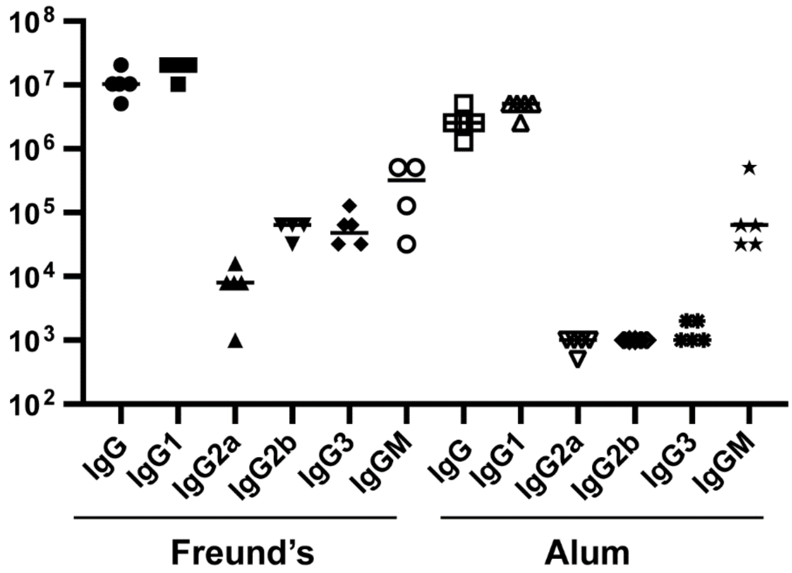

Figure S3. Immunogenicity of NHBA. ELISA titers of the post-immune sera from each mouse immunized with either NHBA-c-Freund's or NHBA-c-Alum against purified recombinant NHBA. The titer for each of five mice are shown with symbols, and the geometric mean titer (GMT) is indicated with a bar.

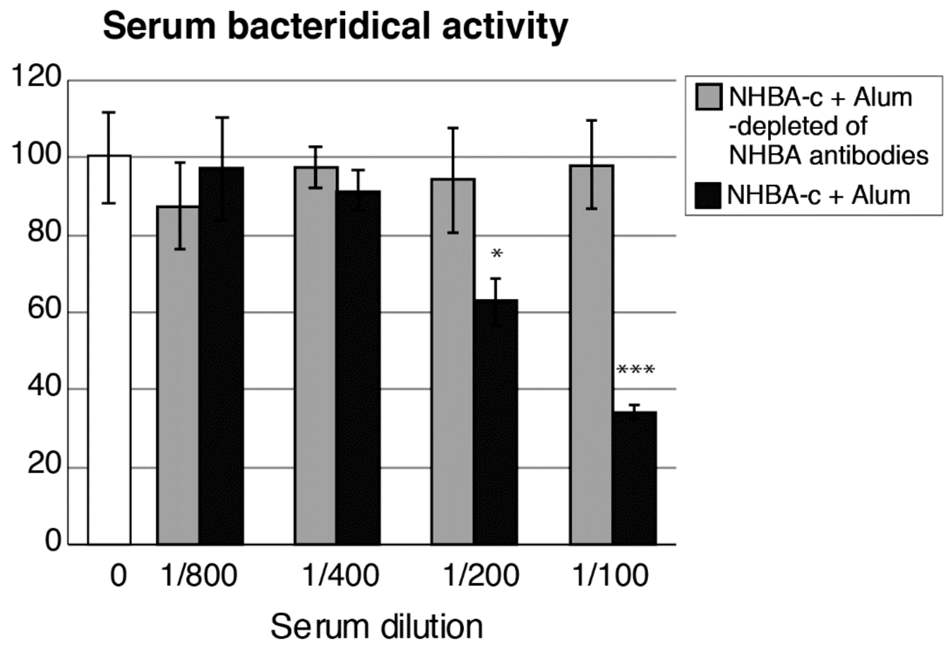

Figure S4. Serum bactericidal activity (SBA) of anti-NHBA serum. The survival of *N. gonorrhoeae* strain 1291 in the presence of normal human serum as a source of complement and 2-fold dilutions of heat-inactivated mouse sera is shown. Sera is either anti-NHBA-c serum plus Alum or anti-NHBA-c serum plus Alum that has been depleted of anti-NHBA antibodies. Data represent the average survival for triplicate samples relative to the result obtained with the untreated wild-type strain (0) (the untreated wild type, set at 100%, represent 3.3 x 10^3 colony forming units). Error bars represent ±1 standard deviation. A two-tailed Student's *t*-test was used to compare survival relative to the untreated wild type shown in white (0); *, $P < 0.05$, ***, $P \leq 0.001$.

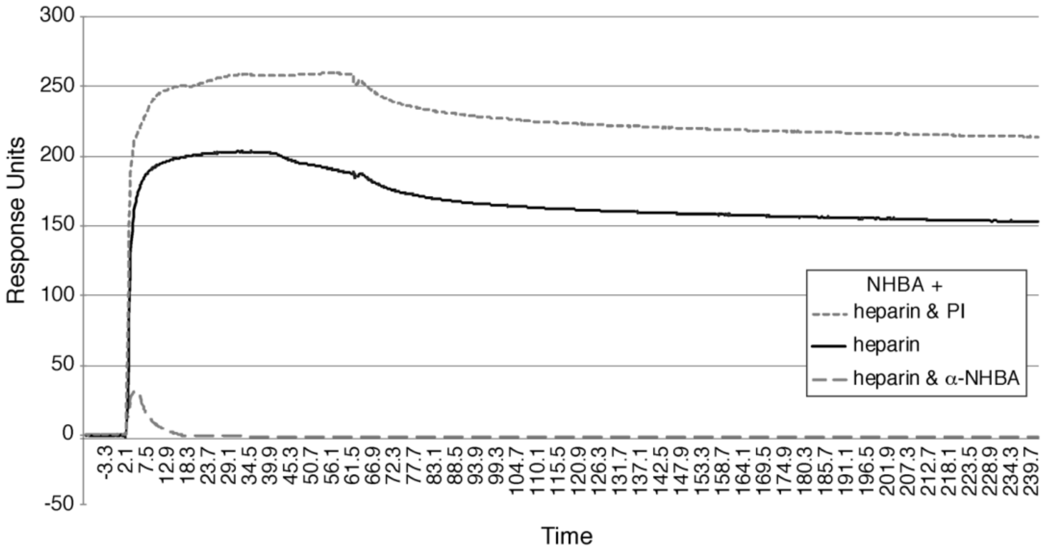

Figure S5. Surface plasmon resonance (SPR) analysis of NHBA-heparin interactions. Representative sensorgrams of SPR analysis of recombinant NHBA binding to heparin in the presence of pre-immune sera (heparin & PI), no sera (heparin) or α-NHBA-Freund's post immune sera (heparin & α-NHBA). Response units are arbitrary units produced due to mass change on the sensor chip across time. Time is in seconds.

Table S1. Distribution of NHBA peptide variants in *N. gonorrhoeae* isolates that have an annotated NHBA protein in the PubMLST database.

NHBA peptide	Frequency	Percentage
542	1407	39.68
475	1078	30.40
481	406	11.45
725	117	3.30
729	117	3.30
543	57	1.61
686	55	1.55
730	55	1.55
737	34	0.96
721	32	0.90
714	32	0.90
731	32	0.90
726	28	0.79
527	18	0.51
722	10	0.28
739	8	0.23
723	6	0.17
724	6	0.17
687	5	0.14
822	4	0.11
685	4	0.11
738	4	0.11
821	3	0.08
720	3	0.08
740	2	0.06
718	2	0.06
9	2	0.06

719	2	0.06
717	2	0.06
480	2	0.06
732	2	0.06
734	1	0.03
735	1	0.03
1252	1	0.03
823	1	0.03
683	1	0.03
733	1	0.03
727	1	0.03
736	1	0.03
824	1	0.03
684	1	0.03
728	1	0.03

© 2020 by the authors. Submitted for possible open access publication under the terms and conditions of the Creative Commons Attribution (CC BY) license (http://creativecommons.org/licenses/by/4.0/).
